# Shiftwork and insulin resistance in professional drivers: exploring the association using non-insulin-based surrogate measures

**DOI:** 10.1186/s12889-024-21243-9

**Published:** 2025-01-16

**Authors:** Mirella Youssef Tawfik, Shaimaa A. A. M. Amer, Ahmed Mahmoud Fouad

**Affiliations:** Department of Public health, Occupational and Environmental Medicine, Faculty of Medicine, Suez Canal University, Ismailia, Egypt

**Keywords:** Shiftwork, Insulin resistance, Non-insulin-based IR surrogates, Professional drivers, Egypt

## Abstract

**Background:**

Previous research has made use of the Homeostatic Model Assessment for Insulin Resistance (HOMA-IR) index to explore the association between shiftwork (SW) and insulin resistance (IR). However, the limitations of the HOMA-IR index restrict its use. This study aimed to investigate the relationship between SW and IR in professional drivers using four alternative non-insulin-based IR surrogate measures (NIRS), and to determine the predictors of elevated NIRS.

**Methods:**

A comparative cross-sectional study was conducted on professional drivers at four Egyptian companies, where 187 SW were compared to 193 dayworkers (DW). Measurements included: sociodemographic, work, and clinical characteristics. Laboratory and NIRS data included: triglyceride glucose (TyG), triglyceride glucose-body mass index (TyG-BMI), triglyceride to high density lipoprotein cholesterol (TG/HDL-C), and metabolic score of insulin resistance (METS-IR). Further assessments included insomnia severity index (ISI), and perceived stress scale (PSS-10).

**Results:**

Shiftwork-drivers showed significantly higher levels of NIRS compared to DW-drivers. Shiftwork was significantly associated with elevated TyG (OR: 5.04, 95% CI: 1.98–12.84), TyG-BMI (OR: 4.50, 95% CI: 2.45–8.26), and METS-IR (OR: 6.30, 95% CI: 2.72–14.58). Significant interactions between SW and insomnia or meal-timing habits existed, where SW-drivers with clinically significant insomnia had 11 times higher odds of elevated TyG compared to DW drivers without insomnia. Likewise, SW-drivers experiencing poor meal timing habits had 5.5- and 6.8-times higher odds of elevated TG/HDL-C and METS-IR, respectively, compared to DW divers without poor meal timing habits. Other significant predictors for elevated NIRS included: age, income, stress, overweight/obesity, and poor meal timing habits.

**Conclusions:**

This study demonstrates a significant association between shiftwork and elevated insulin resistance in professional drivers. Insomnia and poor meal timing habits significantly increases the odds of insulin resistance among professional drivers, suggesting interventions targeting sleep quality, meal timing, and stress management.

**Supplementary Information:**

The online version contains supplementary material available at 10.1186/s12889-024-21243-9.

## Background

The circadian timing system regulates glucose homeostasis, which exhibits circadian rhythmicity with higher insulin sensitivity and levels in the morning than in the evening [1]. Circadian rhythm desynchronization occurs when the circadian timing system’s internal oscillations are out of sync with the daily rhythm of the external environment [2]. About 20% of people involved in shift work (SW) experience this internal-external desynchronization [3]. Shift work refers to a work schedule that is not between the hours of 7 a.m. and 6 p.m [4]. and can include fixed or rotating schedules as well as shifts that are early in the morning, late at night, or in the evening. Drivers are one of the trade unions who are most exposed to SW due to the nature of their job.

Recent studies have confirmed that desynchronization of the circadian rhythm is linked to an increased risk of insulin resistance (IR) and related diseases [2, 5].Previous studies conducted to examine the association between SW and IR by the homeostasis model assessment of IR (HOMA-IR) index proposed that SW may be related to IR [6, 7].However, the low practicality and poor reproducibility of the HOMA-IR index [8], in addition to the requirement for insulin measurement [9]limit the ability of the use of this index in primary clinical practice as well as in epidemiological studies. An ideal alternative solution is the use of IR surrogates that are considered clinically useful for the early identification of IR and can also be easily measured using routine clinical and laboratory measures that are affordable in clinical settings. Numerous non-insulin-based IR surrogates (NIRS) have been the subject of recent studies as novel and clinically valuable surrogates for IR prediction. The triglycerides-glucose index (TyG) [10], the triglycerides-glucose-body mass index (TyG-BMI) [11], the triglycerides-to-high-density lipoprotein cholesterol ratio (TG/HDL-C) [12], and the metabolic score for IR (METS-IR) [13] are some examples of these surrogates.

We have found only one study that looked at the relationship between IR and SW using TG/HDL-C, one of these NIRS, and concluded a positive correlation between the two [14]. Therefore, we thought to examine whether NIRS that comprise other different metabolic components or comprise a combination of both metabolic and anthropometric components would show comparable results. The use of non-invasive NIRS would help provide feasible as well as essential baseline information for health interventions that aim to improve IR in affected population. Also, the exploit of such simple NIRS for determining risk factors that predict IR in professional drivers would help to determine the subgroups that would benefit more from such interventions. Studies that investigated the factors predicting IR in professional drivers are scarce. A recent comparative study conducted on professional drivers concluded that SW, metabolic syndrome, and driving location were associated with increased levels of IR as measured by the HOMA-IR index [7]. Authors in this previous study, however, did not include essential lifestyle repercussions that could be associated with SW and would have impact on IR, (e.g. meal timing habits [15], sleep quality and duration [16], and stress [17, 18]).This study aims to examine the association between SW and IR in professional drivers using four different NIRS, namely TyG, TyG-BMI, TG/HDL-C, and METS-IR, and to identify factors that predict elevated levels of NIRS among them.

## Methods

### Study design, setting and participants

This is a comparative cross-sectional study that was carried out in the occupational health clinics of four Petroleum Companies located in the province of El-Maadi Cairo-Egypt, in the period from May 2023 to the end of February 2024. The criteria for inclusion included full-time professional drivers aged between 22 and 60 years. The lower age limit of 22 years aligns with Egyptian traffic regulations stipulating a minimum driving age of 21 years with at least one year of experience on the current shift. Exclusions from the study included those with a history of diabetes, cardiovascular disease (coronary heart disease, specifically manifested as heart attacks, and cerebrovascular diseases, specifically manifested as strokes), cancer, chronic kidney or liver disease, taking weight-control medications or adhering to a weight-loss program, and working multiple jobs. Every recruited driver received an invitation to participate and an explanation of the purpose and methodology of the study. Study-related data were gathered during the drivers’ regularly scheduled physical examinations, and laboratory work was done in the company related lab.

### Measures and tools

Participants’ self-reported data were collected using a structured face-to-face interview questionnaire [Supplementary File 1] developed specifically for this study. The questionnaire was administered by nurses at the company’s on-site clinics. These nurses underwent specific training on the questionnaire’s design, purpose, and data collection procedures. The questionnaire included both original questions designed by the authors to gather demographic, medical history, family history, work-related, and lifestyle data (i.e., smoking status, physical activity, sleep duration). It also incorporated previously developed and published scales and questions, to assess other lifestyle-related factors, including drivers’ sleep quality, perceived stress, and meal timing habits. Participants’ medical data of having hypertension, and/ or dyslipidemia were defined based on their medical history of being diagnosed with the condition.

Based on their work shift schedules, drivers were categorized into two groups: day work-drivers (DW), who work from 7 a.m. to 6 p.m., and SW-drivers, who work from 6 p.m. to 7 a.m., with a minimum duration of 6 h for both groups. Smoking status was assessed using one question with 2 responses: non-smoker (never smoked/former smoker), and smoker (every-day/someday smoker). Level of physical activity was assessed using one question with a frequency response scale from 0 to 7 days, i.e. “How many days/week do you do moderate/vigorous exercise (e.g., fast walking/running)? Responses were categorized into inactive, moderately active, and very active based on the frequency scale of 0–2, 3–4, and ≥ 5days/week.

Drivers’ sleep quality was assessed over the preceding 2 weeks using the validated Arabic version of the Insomnia Severity Index (ISI) [19]. The ISI includes seven-items rated on a 5- point Likert scale with 0 denoting no insomnia at all and 4 denoting extremely severe insomnia. Four categories of insomnia were derived: no clinically significant insomnia (0–7), sub-threshold insomnia (8–14), moderate clinical insomnia (15–21), and severe clinical insomnia (22–28) [20]. The ISI index had a high internal consistency, with Cronbach’s alpha of 0.87, and a single-factor structure in explanatory factor analysis results that explained 65.7% of the total variance [19]. The duration of sleep was assessed using a single question i.e. “In the preceding 2 weeks, how many hours a day on average did you sleep?”

Perceived stress was assessed using the Arabic Perceived Stress Scale-10 (PSS-10) [21]. This measure evaluates the perceived level of stress over the previous month using 10 questions with a five-point Likert scale ranging from the lowest perceived stress “0” (never) to the highest perceived stress “4” (very often). Perceived stress was categorized to low, moderate, and severe stress for scores ranging from 0 to 13, 14–26, and 27–40 respectively. The Arabic PSS-10 demonstrated adequate validity and reliability [21]. The ISI and PSS-10 scales were transitioned from self-administered to structured interview format. Reliability analysis of the current study sample revealed Cronbach’s alpha of 0.902 for the ISI and 0.887 for the PSS-10.

Meal timing habits were assessed using 3 questions, which have previously been published [22], with responses recalled during the past two weeks and a range from “not once” to “throughout” the week. One question was used to ask about skipping breakfast, i.e. “On average, how many times a week did you skip breakfast? and two questions were used to ask about night eating habits; “On average, how many times per week do you eat “a late dinner before bed”, i.e. between 9 p.m. and 6 a.m.? – we modified the original question to specifically capture eating during late night which is known to be associated with insulin resistance – and “How frequently a week do you eat snacks after dinner?” Drivers were defined to have a “poor” meal timing habit based on a cut-off point of a response of ≥ 3 times/week for each one of the three questions.

Participants’ clinical data included blood pressure, and anthropometric measures of general and central adiposity [body mass index (BMI) and waist circumference (WC) respectively]. The blood pressure was measured using the standard mercury sphygmomanometer in the left arm. The average of two measures (≥ 2 minutes apart) was obtained after the participant had been sitting for ≥ 5 min. Participants’ BMI was computed using their heights and weights [BMI = weight (kg)/height (m^2^)]. Height and weight were measured using the height measuring stand with weighing scale with participants wearing clothing without shoes. Waist circumference was measured to the nearest 0.5 cm in the horizontal plane midway between the lowest ribs and the iliac crest, without compressing the skin at the end of normal expiration.

Participants were subjected to blood sampling after fasting for 8 h. Laboratory data included fasting plasma glucose (FPG), triglycerides (TG), total cholesterol (TC), low density lipoproteins cholesterol (LDL-C), and high-density lipoproteins cholesterol (HDL-C) (all were expressed in mg/dl). The following formulas were used for calculating TyG, TyG-BMI, TG/HDL-C and METS-IR :

- TyG = ln[(TG (mg/dL) × FPG (mg/dL)]/2 [10];
- TyG-BMI = TyG ×BMI [11];
- TG/HDL-C = TG (mg/dL)/HDL-C (mg/dL) [12]; and.
- METS-IR = ln [(2 *FPG (mg/dL)) + TG (mg/dL)] * BMI/ln (HDL-C (mg/dL) [13 ].

### Sampling and sample size justification

A sample size of 182 in each group was estimated to be large enough for testing the association between NIRS and SW using Epi Info™ for Windows (version 7.2.6), for unmatched cross-sectional studies, with 95% CI, power of 80%, ratio of unexposed to exposed = 1, proportion of elevated TG/HDL-C IR surrogate in exposed = 30.5% [14], and an expected odds ratio of 2.0. This number was also large enough to identify significant predictors (X), of at least 1.5 adjusted odds ratio, for the elevated NIRS measure (Y) – at 95% level of confidence, 80% study power, Pr(Y = 1|X = 1) H0 of 0.5, R^2^ other X of 0.40, and a normal distribution of X (µ = 0, σ = 1), where the calculated total sample size was 346 using G*Power 3.1.9.7 for Windows [23].

### Data management and statistical analysis

All statistical analyses were performed with SPSS version 27.0 (IBM Corporation, Armonk, NY, USA). The Kolmogorov Smirnov test showed non-normality of continuous data; thus medians and interquartile ranges (IQR) were used for their presentation, and Mann-Whitney U test was used for testing of between-groups differences. Categorical variables were presented as frequencies and percentages, and univariate associations were evaluated with the Chi-square test. Day work drivers’-derived median values of NIRS were used as sample-based cut-off points to identify participants with elevated NIRS levels (if > median). Accuracy of these cut-off points was assessed using external cut-off points retrieved from related literatures [13, 24, 25]. Logistic regression was performed to determine the adjusted association between elevated NIRS and SW, as well as other potential predictors. The purposeful selection of covariates approach was utilized to identify the best-fit regression model. Covariates were included in the model if they exhibited a *p*-value less than 0.20 in univariate analyses, because conventional p-value levels such as 0.05 can fail in identifying variables known to be important. Since NIRS represent the function of many independent variables (i.e. FPG, TG, BMI, and HDL-C), these variables were excluded from the regression model, as including them as separate variables would create collinearity and potentially inflate variance estimates. Covariates were retained in the model if they if they are significant or a confounder. Confounding is evaluated as a change in the parameter estimate of shiftwork greater than 10% as compared to the full model. Work duration was retained in all models due to its relative importance. Furthermore, all possible interactions between SW and other covariates were tested for statistical significance. The goodness-of-fit of the model was evaluated using Hosmer-Lemeshow test, Nagelkerke R Square, and receiver operating characteristic (ROC) curve analysis. Parameter estimates of the regression models were presented as odds ratios (OR) with corresponding 95% confidence intervals (CI) and statistical significance was set at *p* < 0.05.

## Results

This study included 193 DW and 187 SW professional male drivers. Compared to DW-drivers, SW-drivers exhibited significantly lower physical activity levels (*p* < 0.05), poorer meal timing habits including skipping breakfast and eating dinner late before bed (*p* < 0.001), higher ISI score (*p* < 0.001), higher prevalence of moderate/severe insomnia (76.0% vs. 52.8%, *p* < 0.001), fewer sleeping hours/day (median = 6 and 8 h respectively, *p* < 0.001). Additionally, they had higher WC, BMI, overweight/obesity prevalence (70% vs.57%, *p* < 0.001), and dyslipidemia prevalence (27.3% vs.10.9%, *p* < 0.001) (Table 1). Shift work drivers displayed significantly lower HDL-C, significantly higher FPG, TC, LDL-C, TG, and NIRS, and higher prevalence of elevated NIRS as by using sample-based and external cut-off points, compared to DW drivers (Table 2).

**Table 1:** Self-reported and clinical data of the studied daywork and shiftwork drivers

	Characteristics	Daywork drivers (*n* = 193)	Shiftwork drivers (*n* = 187)	*p*-value
Age (years)	Median (IQR) ^a^	45 (6.0)	46 (9.0)	0.091
Education N (%)	Prim/Prep school	113 (58.5%)	103 (55.1%)	0.495
	Secondary & university	80 (41.5%)	84 (44.9%)	
Marital status N (%)	Single/widow/divorced	17 (8.8%)	13 (7%)	0.502
	Married	176 (91.2%)	174 (93%)	
Average monthly income N (%)	≤ 12,000 EGP	126 (65.3%)	110 (58.8%)	0.194
	> 12,000 EGP	67 (34. %7)	77 (41.2%)	
Comorbidities N (%)	Hypertension	77 (39.9%)	63 (33.7%)	0.210
	Dyslipidemia	21 (10.9%)	51 (27.3%)	0.000**
Relevant Family history ^b^ N (%)		72 (37.3%)	72 (38.5%)	0.810
Type of vehicle N (%)	Car/bus	73(37.8%)	86 (46.0%)	0.107
	Trucks/trailers	135 (69.9%)	101 (54.0%)	
Driving location N (%)	Suburban	83 (43.0%)	92 (49.2%)	0.226
	Urban	110 (57.0%)	95 (50.8%)	
Duration of work (years)	Median (IQR)	12 (5.0)	12 (5.0)	0.602
Working hours per day	Median (IQR)	9 (4.0)	10 (4.0)	0.442
Smoking status N (%)	Never smoker	34 (17.6)	39 (20.9)	0.471
	Former smoker	17 (8.8)	21 (11.2)	
	Current smoker	142 (73.6)	127 (67.9)	
Physical activity level N (%)	Inactive	86 (44.6%)	109 (58.3%)	0.021*
	Moderately active	83 (43.0%)	64 (34.2%)	
	Very active	24 (12.4%)	14 (7.5%)	
Poor meal timing habits N (%)	Skipping breakfast	23 (12%)	56 (29.9%)	0.000**
	Eating dinner late before bed	58 (30.1%)	126 (67.4%)	0.000**
	Having snacks after dinner	37 (19.2%)	32 (17.1%)	0.603
Number of poor meal timing habits N (%)	None	123 (63.7%)	49 (26.2%)	0.000**
	Single	31 (16.1%)	77 (41.2%)	
	Multiple	39 (20.2%)	61 (32.6%)	
Sleeping hours /day	Median (IQR)	8 (2.0)	6 (3.0)	0.000**
Insomnia severity index (ISI) score	Median (IQR)	15 (7.0)	20 (10.0)	0.000**
Insomnia categories N (%)	No clinically significant/Sub-threshold	91 (47.2%)	45 (24.1%)	0.000**
	Moderate	68 (35.2%)	51 (27.3%)	
	Severe	34 (17.6%)	91 (48.7%)	
Perceived stress scale (PSS-10)	Median (IQR)	25 (9.0)	24 (10.0)	0.736
PSS − 10 categories N (%)	Low	20 (10.4%)	22 (11.8%)	0.893
	Moderate	101 (52.3%)	98 (52.4%)	
	Severe	72 (37.3%)	67 (35.8%)	
Blood pressure ^a^ (mmHg)	Systolic	120 (20.0)	120 (20.0)	0.070
	Diastolic	80 (5.0)	80 (5.0)	0.889
Waist circumference (WC)	Median (IQR)	89 (15.0)	95 (12.0)	0.000**
Body mass index (BMI)	Median (IQR)	25 (4.7)	28.1 (5.6)	0.000**
BMI categories N (%)	Normal	83 (43.0%)	56 (29.9%)	0.000**
	Overweight	96 (49.7%)	70 (37.4%)	
	Obese	14 (7.3%)	61 (32.6%)	

^a^.IQR: Interquartile range

^b^.Hypertension, diabetes, atherosclerotic cardiovascular diseases, stroke

*Statistically significant at *p* < 0.05; **Statistically significant at *p* < 0.01

**Table 2:** Laboratory data, non-insulin-based IR surrogates (NIRS) median values and proportions in the studied daywork, and shiftwork drivers

Measures	Daywork drivers (*n* = 193)	Shiftwork drivers (*n* = 187)	*p*-value
**Laboratory data median (IQR)**			
FPG (mg/dl)	95 (11.0)	99 (15.0)	0.000***
Total Cholesterol (mg/dl)	192 (45.0)	200 (59.0)	0.002**
HDL-C (mg/dl)	48 (23.0)	40 (19.0)	0.002**
LDL (mg/dl)	114 (57.0)	123 (71.0)	0.02*
TG (mg/dl)	131 (38.0)	156 (48.0)	0.000***
**NIRS median (IQR)**			
TyG	4.71 (0.19)	4.84 (0.25)	0.000***
TyG-BMI	119.1 (26.8)	135.39 (24.9)	0.000***
TG/HDL-C	2.93 (2.3)	4.09 (2.92)	0.000***
METS-IR	38.07 (9.38)	42.73 (9.45)	0.000***
**N (%) with above NIRS sample-based cut-off points** ^**a**^			
TyG	95 (49.2)	146 (78.1)	0.000***
TyG-BMI	96(49.7)	148 (79.1)	0.000***
TG/HDL-C	97 (50.3)	136 (72.7)	0.000***
METS-IR	97 (50.3)	114 (61.0)	0.000***
**N (%) with above NIRS external cut-off points** ^**b**^			
TyG	133 (68.9)	171 (91.4)	0.000***
TyG-BMI	39 (20.2)	56 (29.9)	0.000***
TG/HDL-C	119 (61.7)	139 (74.3)	0.000***
METS-IR >	18 (9.3)	34 (18.2)	0.002**

^a^4.71, 119.07, 2.93, 38.07 (TyG, TyG-BMI, TG/HDL-C, and METS-IR respectively)

^b^4.66, 135.50, 2.60, 50.93 (TyG, TyG-BMI, TG/HDL-C, and METS-IR respectively)

*Statistically significant at *p* < 0.05,**Statistically significant at *p* < 0.01; ***Statistically significant at *p* < 0.001

Table 3 shows the associations between self-reported and clinical data with NIRS. Older age was significantly associated with all elevated NIRS (*p* < 0.01). Non-married status was significantly associated with elevated METS-IR (*p* = 0.028), while it was not significantly associated with other NIRS. Lower monthly income was significantly associated with elevated TyG, TyG-BMI, TyG/HDL-C (*p* < 0.05), while it did not show significant association with METS-IR (*p* = 0.101). Hypertension and dyslipidemia were significantly associated with all elevated NIRS (*p* < 0.01). Longer work duration and driving location (suburban) were significantly associated with the elevated METS-IR only (*p* = 0.004, and 0.048, respectively). Shiftwork was significantly associated with all elevated NIRS compared to dayshift (*p* < 0.001). Physical inactivity was significantly associated with elevated TyG and TyG/HDL-C compared to physical activity, while did not show significant association with TyG-BMI or METS-IR. Moreover, poor meal timing habits; lower number of sleep hours per day; and higher insomnia severity, perceived stress, systolic blood pressure, WC, and WC; and overweight or obesity were significantly associated with all elevated NIRS (*p* < 0.01).

**Table 3:** Association of self-reported and clinical data with elevated NIRS using NIRS sample-based cut-off points: results of Univariate analysis (*N* = 380)

Study variables	TyG			TyG-BMI			TyG/HDL-C			METS-IR		
	Elevated (n=241)	Normal (n=139)	*p-value*	Elevated (n = 244)	Normal (n=136)	*p-value*	Elevated (n = 233)	Normal (n = 147)	*p-value*	Elevated (n = 247)	Normal (n = 133)	*p-value*
**Age (years)** ^a^	47 (6.0)	45 (8.0)	0.005**	47 (7.0)	45 (7.0)	0.000***	47 (6.0)	45 (8.0)	0.000***	47 (6.0)	45 (7.0)	0.000***
**Education N (%)**												
Prim/Prep school	140 (58.1)	76 (54.7)	0.517	143 (58.6)	73 (53.7)	0.352	141 (60.5)	75 (51.0)	0.069	142 (57.5)	74 (55.6)	0.728
Secondary & university	101 (41.9)	63 (45.3)		101 (41.4)	63 (46.3)		92 (39.5)	72 (49.0)		105 (42.5)	59 (44.4)	
**Marital status N (%)**												
Not married	23 (9.5)	7 (5.0)	0.117	24 (9.8)	6 (4.4)	0.060	22 (9.4)	8 (5.4)	0.159	25 (10.1)	5 (3.8)	0.028*
Married	218 (90.5)	132 (95.0)		220 (90.2)	130 (95.6)		211 (90.6)	139 (94.6)		222 (89.9)	128 (96.2)	
**Average monthly income N (%)**												
≤ 12000 EGP	140 (58.1)	96 (69.1)	0.034*	141 (57.8)	95 (69.9)	0.020*	128 (54.9)	108 (73.5)	0.000***	146 (59.1)	90 (67.7)	0.101
> 12000 EGP	101 (41.9)	43 (30.9)		103 (42.2)	41 (30.1)		105 (45.1)	39 (26.5)		101 (40.9)	43 (32.3)	
**Comorbidities N (%)**												
Hypertension	102 (42.3)	38 (27.3)	0.004**	109 (44.7)	31 (22.8)	0.000***	106 (45.5)	34 (23.1)	0.000***	107 (43.3)	33 (24.8)	0.000***
Dyslipidemia	56 (23.2)	16 (11.5)	0.005**	57 (23.4)	15 (11.0)	0.003**	58 (24.9)	14 (9.5)	0.000***	58 (23.5)	14 (10.5)	0.002**
**Relevant Family history N (%)**												
No	149 (61.8)	87 (62.6)	0.882	146 (59.8)	90 (66.2)	0.222	139 (59.7)	97 (66.0)	0.215	147 (59.5)	89 (66.9)	0.156
Yes	92 (38.2)	52 (37.4)		98 (40.2)	46 (33.8)		94 (40.3)	50 (34.0)		100 (40.5)	44 (33.1)	
**Type of vehicle N (%)**												
Car/bus	98 (40.7)	61 (43.9)	0.540	111 (45.5)	48 (35.3)	0.053	93 (39.9)	66 (44.9)	0.337	107 (43.3)	52 (39.1)	0.426
Trucks/trailers	143 (59.3)	78 (56.1)		133 (54.5)	88 (64.7)		140 (60.1)	81 (55.1)		140 (56.7)	81 (60.9)	
**Driving location N (%)**												
Suburban	112 (46.5)	63 (45.3)	0.829	121 (49.6)	54 (39.7)	0.064	113 (48.5)	62 (42.2)	0.229	127 (51.4)	48 (36.1)	0.004**
Urban	129 (53.5)	76 (54.7)		123 (50.4)	82 (60.3)		120 (51.5)	85 (57.8)		120 (48.6)	85 (63.9)	
**Duration of work (years)** ^a^	12 (5.0)	12 (5.0)	0.795	12 (5.0)	12 (5.0)	0.093	12 (5.0)	12 (5.0)	0.978	12 (5.0)	12 (4.0)	0.048*
**Working hours per day** ^a^	8 (4.0)	10 (4.0)	0.330	8 (4.0)	10 (4.0)	0.277	9 (4.0)	9 (4.0)	0.871	9 (4.0)	9 (4.0)	0.410
**Type of work shifts N (%)**												
Day work	95 (39.4)	98 (70.5)	0.000***	96 (39.3)	97 (71.3)	0.000***	97 (41.6)	96 (65.3)	0.000***	97 (39.3)	96 (72.2)	0.000***
Shift work	146 (60.6)	41 (29.5)		148 (60.7)	39 (28.7)		136 (58.4)	37 (34.7)		150 (60.7)	37 (27.8)	
**Smoking status N (%)**												
Never smoker	50 (20.7)	23 (16.5)	0.257	53 (21.7)	20 (14.7)	0.120	45 (19.3)	28 (19.0)	0.414	48 (19.4)	25 (18.8)	0.468
Former smoker	20 (8.3)	18 (12.9)		27 (11.1)	11 (8.1)		27 (11.6)	11 (7.5)		28 (11.3)	10 (7.5)	
Current smoker	171 (71.0)	98 (70.5)		164 (67.2)	105 (77.2)		161 (69.1)	108 (73.5)		171 (69.2)	98 (73.7)	
**Physical activity level N (%)**												
Inactive	137 (56.8)	58 (41.7)	0.007**	133 (54.5)	62 (45.6)	0.224	136 (58.4)	59 (40.1)	0.000***	133 (53.8)	62 (46.6)	0.249
Moderately active	79 (32.8)	68 (48.9)		87 (35.7)	60 (44.1)		74 (31.8)	73 (49.7)		88 (35.6)	59 (44.4)	
Very active	25 (10.4)	13 (9.4)		24 (9.8)	14 (10.3)		23 (9.9)	15 (10.2)		26 (10.5)	12 (9.0)	
**Number of poor meal timing habits N (%)**												
None	57 (23.7)	115 (82.7)	0.000***	72 (29.5)	100 (73.5)	0.000***	59 (25.3)	113 (76.9)	0.000***	71 (28.7)	101 (75.9)	0.000***
Single	92 (38.2)	16 (11.5)		79 (32.4)	29 (21.3)		87 (37.3)	21 (14.3)		87 (35.2)	21 (15.8)	
Multiple	92 (38.2)	8 (5.8)		93 (38.1)	7 (5.1)		87 (37.3)	13 (8.8)		89 (36.0)	11 (8.3)	
**Sleeping hours /day** ^a^	6 (3.0)	7 (2.0)	0.000***	6.5 (3.0)	8 (2.0)	0.000***	6 (3.0)	8 (2.0)	0.000***	7 (3.0)	8 (2.0)	0.002**
**ISI score** ^a^	21 (9.0)	12 (5.0)	0.000***	19 (10.0)	14 (6.0)	0.000***	20 (9.0)	13 (5.0)	0.000***	19 (10.0)	14 (7.0)	0.000***
**Insomnia categories N (%)**												
No clinically significant	37 (15.4)	99 (71.2)	0.000***	54 (22.1)	82 (60.3)	0.000***	37 (15.9)	99 (67.3)	0.000***	54 (21.9)	82 (61.7)	0.000***
Moderate/severe	204 (84.6)	40 (28.8)		190 (77.9)	54 (39.7)		196 (84.1)	48 (32.7)		193 (78.1)	51 (38.3)	
**PSS- 10 score** ^a^	26 (8.0)	21 (11.0)	0.000***	26 (9.0)	22 (9.0)	0.000***	26 (9.0)	23 (10.0)	0.000***	26 (8.0)	23 (10.0)	0.000***
**PSS- 10 categories N (%)**												
Low/Moderate	123 (51.0)	118 (84.9)	0.000***	124 (50.8)	117 (86.0)	0.000***	123 (52.8)	118 (80.3)	0.000***	136 (55.1)	105 (78.9)	0.000***
Severe	118 (49.0)	21 (15.1)		120 (49.2)	19 (14.0)		110 (47.2)	29 (19.7)		111 (44.9)	28 (21.1)	
**Blood pressure (mmHg)** ^a^												
Systolic	120 (10.0)	120 (20.0)	0.000***	120 (12.5)	120 (15.0)	0.000***	120 (10.0)	120(20.0)	0.000***	120(15.0)	120 (20.0)	0.007**
Diastolic	80 (5.0)	80 (5.0)	0.558	80 (5.0)	80 (5.0)	0.014*	80 (5.0)	80 (5.0)	0.423	80 (10.0)	80 (5.0)	0.934
**WC** ^a^	97 (10.0)	87 (9.0)	0.000***	96 (10.0)	85.5 (10.0)	0.000***	98 (10.0)	86 (9.0)	0.000***	96 (10.0)	85 (9.0)	0.000***
**BMI** ^a^	27.8 (4.9)	24.3 (4.3)	0.000***	28.1 (3.6)	22.8 (2.8)	0.000***	27.5 (4.6)	24.3 (5.0)	0.000***	28.1 (3.4)	22.8 (2.8)	0.000***
**BMI categories N (%)**												
Normal	63 (26.1)	76 (54.7)	0.000***	23 (9.4)	116 (85.3)	0.000***	61 (26.2)	78 (53.1)	0.000***	33 (13.4)	106 (79.7)	0.000***
Overweight/Obese	178 (73.9)	63 (45.3)		222 (90.6)	20 (14.7)		172 (73.8)	69 (46.9)		214 (88.6)	27 (20.3)	

^a^ Median (IQR). PSS: Perceived stress scale. BMI: Body Mass Index. WC: Waist circumference. ISI: Insomnia severity index

*Statistically significant at *p* < 0.05, **Statistically significant at *p* < 0.01; ***Statistically significant at *p* < 0.001

The best-fit multiple logistic regression models (based on sample cut-off points) for identifying predictors of NIRS among the study participants were presented in Table 4. Strong significant predictors of elevated TyG included SW, poor meal timing habits, and moderate/severe insomnia (OR 5.04; 95% CI 1.98–12.84; 6.89; 95% CI 3.54–13.75; 12.07; 95% CI 5.23–27.85 respectively). Moreover, a notable interaction between SW and moderate/severe insomnia was found (OR 0.18, 95% CI: 0.05–0.63), indicating that SW-drivers with moderate/severe insomnia had 11 times higher odds of elevated TyG compared to DW drivers without moderate/severe insomnia. Variables showed significant prediction of elevated TyG-BMI included SW, severe perceived stress, moderate/severe insomnia, and hypertension (OR 4.50, 95% CI: 2.45–8.26; 4.70, 95% CI: 2.37–9.29; 2.46, 95% CI: 1.38–4.36; and 2.90, 95% CI: 1.53–5.51, respectively).Variables showed significant prediction of elevated TG/HDL-C included monthly income above 12k LE, overweight/obesity, poor meal timing habits, and moderate/severe insomnia (OR 2.47; 95% CI: 1.21–5.07; 2.25; 95% CI: 1.27–2.98; 2.51, 95% CI: 1.12–5.64; and 2.58, 95% CI: 2.56–8.20, respectively). A notable interaction between SW and poor meal timing habits was found (OR 3.34; 95% CI 1.02–10.95) indicating that SW-drivers experiencing poor meal timing habits had 5.5 times higher odds of elevated TG/HDL-C compared to DW divers without poor meal timing habits. Variables showed significant prediction of elevated METS-IR included SW, age, poor meal timing habits, and moderate/severe insomnia (OR 6.30; 95% CI 2.72–14.58; 8%; 95% CI 1.01–1.16; 4.09; 95% CI 2.17–7.71; 4.94; 95% CI 2.41–10.10). Moreover, an interaction between SW and moderate/severe insomnia was identified (OR 0.22; 95% CI 0.07–0.66), indicating that SW-drivers with clinically significant insomnia had 6.8 times higher odds of elevated METS-IR compared to DW drivers without clinically significant insomnia. As shown in the Table S1 (Supplementary File 2), replication of the above regression models using external criteria cut-off points for determining elevated NIRS revealed similar findings, with no detected statistically significance in difference between each model and its counterpart using ROC analysis (Table S2, Figure S1, Supplementary File 2).

**Table 4:** Variables predicting elevated NIRS using sample-based median cut-off points (*N* = 380): results of multivariate analyses

IR Surrogate	Predictors	OR	95% CI	*p*-value	Model fit
**TyG**	**Shiftwork (vs. daywork)**	**5.04**	**1.98 – 12.84**	0.000***	Overall accuracy: 82.6% Hosmer & Lemeshow Test: Χ^2^=7.2, df=8, *p*-value=0.516 Nagelkerke R Square: 0.564 AUC=0.898
	Age (years)	1.02	0.95 – 1.10	0.561	
	Physical activity (Moderately active vs. inactive)	1.02	0.53 – 1.95	0.957	
	Physical activity (Very active vs. inactive)	1.18	0.45 – 3.06	0.736	
	Overweight/obese (vs. normal weight)	1.82	0.99 – 3.34	0.055	
	Work duration (years)	0.94	0.85 – 1.04	0.209	
	Poor meal timing habits (single/multiple vs. none)	6.89	3.54 – 13.75	0.000***	
	PSS-10 (severe vs. low stress)	2.01	0.98 – 4.11	0.057	
	Moderate/severe insomnia (vs. No clinically significant/Sub-threshold)	12.07	5.23 – 27.85	0.000***	
	**Shiftwork-by-moderate/severe insomnia**	0.18	0.05 – 0.63	0.007**	
	*Model Constant*	0.06	-	0.045*	
**TyG-BMI**	**Shiftwork (vs. daywork)**	**4.50**	**2.45 – 8.26**	0.000***	Overall accuracy: 76.6% Hosmer & Lemeshow Test: Χ^2^=9.80, df=8, *p*-value=0.280 Nagelkerke R Square: 0.41 AUC= 0.816
	Age (years)	1.01	0.95 – 1.08	0.713	
	Physical activity (Moderately active vs. inactive)	1.51	0.85 – 2.68	0.166	
	Physical activity (Very active vs. inactive)	1.38	0.59 – 3.24	0.465	
	Work duration (years)	1.02	0.93 – 1.12	0.642	
	Poor meal timing habits (single/multiple vs. none)	1.82	0.98 – 3.36	0.057	
	PSS-10 (severe vs. low stress)	4.70	2.37 – 9.29	0.000***	
	Moderate/severe insomnia (vs. No clinically significant/Sub-threshold)	2.46	1.38 – 4.36	0.002**	
	Hypertension	2.90	1.53 – 5.51	0.001*	
	*Model Constant*	0.07	-	0.033*	
**TG/HDL-C**	Shiftwork (vs. daywork)	0.66	0.29 – 1.48	0.310	Overall accuracy: 80.3% Hosmer & Lemeshow Test: Χ^2^=4.34, df=8, *p*-value=0.825 Nagelkerke R Square: 0.481 AUC=0.853
	Age (years)	1.02	0.95 -1.10	0.578	
	Average monthly income (>12k vs. ≤12k EGP)	2.47	1.21 – 5.07	0.014*	
	Physical activity (Moderately active vs. inactive)	0.81	0.45 – 1.47	0.491	
	Physical activity (Very active vs. inactive)	0.94	0.38 – 2.30	0.890	
	Overweight/obese (vs. normal weight)	2.25	1.27 – 2.98	0.005**	
	Work duration (years)	0.92	0.84 – 1.01	0.091	
	Poor meal timing habits (single/multiple vs. none)	2.51	1.12 – 5.64	0.026*	
	PSS-10 (severe vs. low stress)	1.20	0.63 – 2.30	0.581	
	Moderate/severe insomnia (vs. No clinically significant/Sub-threshold)	4.58	2.56 – 8.20	0.000***	
	**Shiftwork-by-bad meal timing habits**	**3.34**	**1.02 – 10.95**	0.046*	
	*Model Constant*	0.172	-	0.237	
**METS-IR**	**Shiftwork (vs. daywork)**	**6.30**	**2.72 – 14.58**	0.000***	Overall accuracy: 77.4% Hosmer & Lemeshow Test: Χ^2^=5.54, df=8, *p*-value=0.698 Nagelkerke R Square: 0.393 AUC=0.826
	Age (years)	1.08	1.01 – 1.16	0.032*	
	Average monthly income (>12k vs. ≤12k EGP)	0.66	0.34 – 1.28	0.214	
	Physical activity (Moderately active vs. inactive)	1.63	0.90 – 2.94	0.104	
	Physical activity (Very active vs. inactive)	1.35	0.56 – 3.24	0.503	
	Work duration (years)	1.04	0.95 – 1.14	0.401	
	Poor meal timing habits (single/multiple vs. none)	4.09	2.17 – 7.71	0.000***	
	PSS-10 (severe vs. low stress)	1.51	0.81 – 2.83	0.198	
	Moderate/severe insomnia (vs. No clinically significant/Sub-threshold)	4.94	2.41 – 10.10	0.000***	
	**Shiftwork-by-moderate/severe insomnia**	0.22	0.07 – 0.66	0.007**	
	*Model Constant*	0.004	-	0.000***	

*Statistically significant at *p* <0.05,** Statistically significant at *p* < 0.01; ***Statistically significant at *p* < 0.001

## Discussion

The present study aimed at evaluating the association between different NIRS and SW among professional drivers and determining the variables predicting elevated levels of NIRS among them. It is important to note that the companies participating in this study employed work shift systems that exclusively comprised fixed day shifts (7am-6pm) and fixed night shifts (6pm-7am). As such, rotational SW, characterized by regular changes in shift schedules, was not represented in the study population. Previous research has consistently linked rotational shift work to an elevated risk of IR, primarily attributed to night shift induced disruptions in circadian rhythm [26]. By focusing on the comparison of fixed day and SW encompassing night shifts, our study aims to isolate the core impact of SW on NIRS, un-confounded by the complexities of rotational schedules.

The descriptive statistics in our study revealed inconsistency in lifestyle, anthropometric, and metabolic characteristics between DW and SW-drivers, which suggested the potential influence of these underlying characteristics on NIRS. It is noteworthy that the day worker group in our study also exhibited suboptimal metabolic parameters, including lower than recommended HDL levels and a high prevalence of overweight/obesity. It seems that regardless of the shift type, the work conditions of professional drivers make a healthy lifestyle difficult to follow. In our study, the significant association between the four examined NIRS and SW confirmed our expectation regarding the hypothesized relationship between them. Theoretically, the four examined NIRS represent the function of the metabolic and the anthropometric components (i.e. FPG, TG, HDL-C, and BMI respectively) which were shown in our univariate analysis to have significant association with SW. Studies that examined the association between SW and NIRS are scarce. One study has examined the association between SW and TG/HDL-C among apparently healthy adults and showed significant association which supports results in our study [14]. The higher proportion of the elevated TG/HDL-C (based on both sample as well as external cut-off points) in our study compared to the proportion in this previous study is mainly due to our preference to use lower cut-off points as we were focusing on identifying most of at-risk individuals.

In our study, many lifestyle-related variables that were found to be significantly associated with SW, (i.e. poor sleep quality/duration, poor meal timing habits, and low physical activity) were also found to be significantly associated (in univariate analysis) with TG/HDL-C as well as the other three examined NIRS, which may indicate a mediating role of these variables between SW and NIRS. The significant association of these specific variables with NIRS in our study has also been found to support results from retrieved studies. Studies conducted on non-diabetic population showed significant association between poor sleep quality and high TyG index, and between poor sleep quality and abnormal sleep duration, and TyG-BMI [27], as well as between short sleep duration and high TG/HDL-C [28].A study conducted on non-diabetic Koreans found that lower weekly breakfast consumption was associated with a high TyG [29].Other studies showed that late dinner was associated with increased triglyceride levels, BMI [30, 31], as well as high blood glucose levels [32], which theoretically supports the hypothesis of an association between late dinner and the TyG, TyG-BMI, METS-IR surrogates since the calculation of these surrogates depends on the function of ≥ two of these components.

In our study, results of univariate analysis showed that some variables shared each other for being statistically significant across the four studied NIRS. These variables included: type of work shift, age, BMI, WC, presence of more than one bad food timing habits, hypertension, systolic blood pressure, dyslipidemia, severe perceived stress, ISI score, and sleeping hours. Previous studies had examined the association of some of these variables with one or more NIRS included in our study and showed consistent results. A previous study conducted in Jordan on apparently healthy employees showed a significant positive association between age and the examined NIRS, i.e. TG/HDL-C [14]. Waist circumference was found to be significantly associated with two examined NIRS, i.e. TG/HDL-C and TyG in a study conducted on non-diabetic normal weight adults of both genders in Peru, while BMI showed significant association with TG/HDL-C only in the same study [33]. The presence of significant association between WC and both TyG and TG/HDL-C in our study as well as in this latter study could be due to the nature of visceral adiposity which was previously known to be more associated with dyslipidemia and dysglycemia compared to general adiposity, with a resultant hypertriglyceridemia, reduced HDL-C and high blood glucose levels [34]. The absence of the association between BMI and TyG in the latter study could be attributed to; firstly, the restricted inclusion of participants with a normal BMI category range might have a weak effect on TyG compared to the effect of including a wider BMI categories range. Secondly, the combining of both genders in the later study might dilute the effect of BMI on TyG if the effect is stronger in one gender (the authors of this previous study did not account for gender stratification in studying the association). Thirdly, the use of different cut-off points across studies might have placed most participants in a range where the association of TyG with BMI is weak. Additionally, racial differences among studies might have distinct patterns of central fat distribution, genetic predisposition to insulin sensitivity, and environmental factors that impact results on the association between BMI and TyG.

Since the increase in TG and the decrease in HDL-C constitute pivotal components of dyslipidemia, it is not surprising that the four examined NIRS in our study showed significant associations with dyslipidemia. Previous studies that examined the association between hypertension and NIRS among the general population showed consistent results with our study [35, 36]. Most striking finding in these previous studies as well as in our study was that hypertension showed significant association with all examined NIRS. As TG is the common and constant component in every examined NIRS, the association between hypertension and NIRS could be explained by hypertriglyceridemia inducing IR [37], with a consequently rise in blood glucose level, body fluid volume and hypertension [38]. Also, IR-related hyperinsulinemia may affect the activity of sympathetic nervous system [39], or increase the activation of the renin-angiotensin-aldosterone system, resulting in hypertension [40].

We could not find studies that assessed the association between PSS-10 scores/severity, as well as level of physical activity and NIRS. A previous study that examined the association between stress and IR concluded a negative impact of chronic exposure to stress on serum glucocorticoid concentrations and catecholamine release, which disturb glucose homeostasis, leading to chronic hyperglycemia, increase in insulin requirement and IR [41]. In the same context, physical activity has been found to have an inverse relationship with IR in a study conducted on population without diabetes [42].

The multivariate analysis results in the current study demonstrated that insomnia, and SW (by itself or in conjunction with poor meal timing habits, or severity of insomnia) were the common variables that showed significant prediction (albeit with different odds) of the elevated four NIRS in both predicting models. In addition, a different combination of other variables, such as age, income, PSS-10 severity, overweight/obesity, and poor meal timing habits, were also found to predict NIRS. The performance of “insomnia and SW” throughout the analysis suggest that these variables were strong and consistent predictors of NIRS, and that the link between these two variables and NIRS is independent of the specific thresholds used to define the surrogates’ levels as “elevated”, given that these two models were based on two distinct cut off points. Our analysis showed that the interaction between SW and poor meal timing habits/severity of insomnia showed either an “emerging predicting effect” or an “exacerbating predicting effect” on NIRS. This suggests that maintaining consistent meal schedules and/handling insomnia might be particularly important for individuals engaged in SW to mitigate potential negative effects on their IR. We also found that insomnia (in regression model I) and poor meal timing habits (in regression model II) exhibited the highest statistically significant odds of elevated TyG across the two models (12.07, and 12.63 respectively; *p* < 0.001), which indicates that these variables showed the strongest positive association with TyG. Although the wide range of CI related to these odds (5.23–27.85; and 4.11–38.79 respectively) adds some uncertainty in how strong this association truly is, the association is still strong even at its lowest confidence limit.

Literatures that study the variables predicting high NIRS levels are scarce. We only found one study that examined the ability of poor sleep patterns to predict elevated levels of TyG and TyG-BMI in non-diabetic adults. Results of this previous study, which was conducted with a different methodology, (i.e. on population with diverse races, using distinct sleep measure, alternative NIRS cut-off points, and different covariates in the predicting model), revealed that individuals with trouble sleeping had high odds of having elevated levels of both TyG and TyG-BMI [27]. It is worth noting that, in our study, we did not account for insomnia medication history, which could potentially affect the interpretation of the relationship between sleep quality and NIRS due to the adverse metabolic impact they exert [43, 44]. Given the potential risks associated with these medications in the petroleum industry, we opted not to inquire about their use, especially considering participants were evaluated by medical professionals in a controlled clinical setting.

Shift work and age were also found to be associated with high TG/HDL-C ratio in a study that examined the association of different variables with TG/HDL-C in apparently healthy individuals using multiple linear regressions [14]. Frequency of skipping breakfast and obesity were found in a previous study conducted on non-diabetic Koreans to be associated with higher odds of TyG [28]. While no studies were identified to allow for proper comparison, the likelihood for high NIRS in individuals with high PSS-10 score/severity, and high income found here are consistent with broader trends in health research [41, 45].

In our study, results of ROC curve analysis revealed that the two predicting models perform equally well in classifying individuals which suggests that both models possess similar discriminatory ability. Also, the two models identified quite similar predicting variables, aligning with established knowledge, which suggests the potential of the median cut-off approach for our population.

This study is not without limitations. The difficulty participants encountered in accurately recalling their precise driving hours, making it challenging to categorize shifts into specific timeframes (Truck and trailer drivers struggle to accurately record their driving hours due to mandatory, lengthy, and unscheduled stops between 6pm and 7am to obtain city security permits). Consequently, we were unable to comprehensively examine the influence of shift timing (evening, overnight, early morning) on NIRS results. Second, limited resources forced recruitment from a subset of companies, potentially affecting the generalizability of findings to the broader professional driver population. Third, self-reported data in our study could be influenced by recall/social desirability biases. Fourth, our study has a cross-sectional design with the inability to establish causal inference or temporal relationship.

## Conclusion

This study demonstrates a significant association between shiftwork and elevated insulin resistance in professional drivers, using non-insulin-based surrogate measures (NIRS). Shiftworking drivers exhibited higher levels of triglyceride glucose (TyG), triglyceride glucose-body mass index (TyG-BMI), and metabolic score of insulin resistance (METS-IR) compared to day-working drivers. Moreover, shift working drivers with clinically significant insomnia and poor meal timing habits showed markedly higher odds of elevated NIRS. Other significant predictors for elevated NIRS included age, income, stress, and overweight/obesity. These findings suggest that shiftwork, along with other sociodemographic and lifestyle factors, increases the odds of insulin resistance among professional drivers. Interventions targeting sleep quality, meal timing, and stress management may be crucial in mitigating the risk of insulin resistance among shift working drivers. Future research is needed to elucidate the potential role of mediating/moderating variables on observed relationships.

## Electronic supplementary material

Below is the link to the electronic supplementary material.

Supplementary Material 1

Supplementary Material 2

Supplementary Material 3

Supplementary Material 4

Supplementary Material 5

Supplementary Material 6

## Data Availability

The datasets analyzed during the current study are available from the corresponding author on reasonable request.
