# Supplementary material for: Shiftwork and insulin resistance in professional drivers: exploring the association using non-insulin-based surrogate measures
Source: BMC Public Health. 2025 Jan 16;25:191. doi: 10.1186/s12889-024-21243-9 (PMC11740691; doi:10.1186/s12889-024-21243-9)
Supplement: Supplementary file 1 — Supplementary Material 1 [file 12889_2024_21243_MOESM1_ESM.pdf]

Driver Insulin Resistance Surrogate interview questionnaire

Good morning, thank you for participating in this interview. This research aims to understand factors that might be associated with insulin resistance in professional drivers. All your responses will be kept confidential. The interview will cover the following topics: Demographics, work-related data, medical and family history, and lifestyle habits.

Please answer each question to the best of your ability.

Demographic information

1. How old are you?
2. What is your highest level of education?
   - Primary - preparatory - secondary - university
3. What is your current marital status?
   - Married - single - widow - divorced
4. On average, what is your monthly income?

Work-related information

5. What type of work shift do you typically work?
   - Day shift (7 am - 6 pm) - Night shift (6 pm - 7 am)
6. What kind of vehicle do you primarily drive?
   - Car/bus - trucks/trailers
7. Where do you mainly drive?
   - In suburban locations - in urban locations
8. For how many years have you been working as a professional driver
9. On average, how many hours do you work per day?

Medical information

10. Have you ever been diagnosed with or treated for high blood pressure?
    - Yes - No
11. Have you ever been diagnosed with or treated for high cholesterol or other blood fat problems?
    - Yes - No

Family history information

12. Has either of your parents ever been diagnosed with high blood pressure, diabetes, heart disease, or stroke?

- Yes - No

Lifestyle-related information

13. Have you ever smoked cigarettes or waterpipe?

- Yes - No

14. If yes, how often do you currently smoke?

- Every day - Some days - I quit

15. How many days per week do you typically engage in moderate or vigorous exercise (e.g., fast walking/running)?

- Inactive (0-2 days/week) - moderately active (3-4 days/week) - very active (≥5days/week)

16. Over the past two weeks, on average, how many hours of sleep did you get per night?

Other lifestyle-related information

- **Sleep quality [Insomnia Severity Index (ISI)]**

This brief interview aims to assess your sleep patterns over the past two weeks. All your responses will be kept confidential. Please answer each question considering your sleep quality over the PAST TWO WEEKS. For each question, I will assign a score based on your answer. There is no right or wrong answers, just be honest about your sleep experiences.

Questions:

1. **How difficult is it for you to fall asleep at night?**
   - 0 - Not difficult at all
   - 1 - Somewhat difficult
   - 2 - Moderately difficult
   - 3 - Very difficult
   - 4 - Extremely difficult
2. **How difficult is it for you to stay asleep?**
   - 0 - Not difficult at all

- 1 - Somewhat difficult
- 2 - Moderately difficult
- 3 - Very difficult
- 4 - Extremely difficult

3. **How difficult is it for you to wake up too early?**

- 0 - Not difficult at all
- 1 - Somewhat difficult
- 2 - Moderately difficult
- 3 - Very difficult
- 4 - Extremely difficult

4. **How SATISFIED/DISSATISFIED are you with your CURRENT sleep pattern?**

- 0 - Very satisfied
- 1 - Satisfied
- 2- Moderately satisfied
- 3- Dissatisfied
- 4- Very dissatisfied

5. **How NOTICEABLE to others do you think your sleep problem is in terms of impairing the quality of your life?**

- 0 - Not at all noticeable
- 1- A little
- 2 - Somewhat
- 3 - Much
- 4 - Very much noticeable

6. **How WORRIED/DISTRESSED are you about your current sleep problem?**

- 0 - Not at all worried
- 1- A little
- 2 - Somewhat
- 3 - Much
- 4 - Very much worried

7. **To what extent do you consider your sleep problem to INTERFERE with your daily functioning (e.g. daytime fatigue, mood, ability to function at work/daily chores, concentration, memory, mood, etc.) CURRENTLY?**

- 0 - Not at all interfering
- 1 - A little
- 2 - Somewhat
- 3 - Much
- 4 - Very much interfering

- **Perceived stress [Perceived Stress Scale-10 (PSS-10)]**

This brief interview will assess your stress levels over the past month. All your responses will be kept confidential. For each question, I will read the statement aloud and ask you to consider how often it applied to you over the past month. Please respond by choosing the number that best reflects your experience: 0 – Never, 1 - Hardly ever, 2 – Sometimes, 3 – Often, 4 - Very often

Please answer honestly based on your own experiences.

Questions

l. In the last month, how often have you been upset because of something that happened unexpectedly?

0 – Never 1 - Hardly ever 2 – Sometimes 3 – Often 4 - Very often

2. In the last month, how often have you felt that you were unable to control the important things in your life?

0 – Never 1 - Hardly ever 2 – Sometimes 3 – Often 4 - Very often

3. In the last month, how often have you felt nervous and stressed?

0 – Never 1 - Hardly ever 2 – Sometimes 3 – Often 4 - Very often

4. In the last month, how often have you felt confident about your ability to handle your personal problems?

0 – Never 1 - Hardly ever 2 – Sometimes 3 – Often 4 - Very often

5. In the last month, how often have you felt that things were going your way?

0 – Never 1 - Hardly ever 2 – Sometimes 3 – Often 4 - Very often

6. In the last month, how often have you found that you could not cope with all the things that you had to do?

0 – Never 1 - Hardly ever 2 – Sometimes 3 – Often 4 - Very often

7. In the last month, how often have you been able to control irritations in your life?

   0 – Never 1 - Hardly ever 2 – Sometimes 3 – Often 4 - Very often

8. In the last month, how often have you felt that you were on top of things?

   0 – Never 1 - Hardly ever 2 – Sometimes 3 – Often 4 - Very often

9. In the last month, how often have you been angered because of things that happened and were outside of your control?

   0 – Never 1 - Hardly ever 2 – Sometimes 3 – Often 4 - Very often

10. In the last month, how often have you felt difficulties were piling up so high that you could not overcome them?

    0 – Never 1 - Hardly ever 2 – Sometimes 3 – Often 4 - Very often

- **Meal timing habits**

Next, I will ask you 3 questions about your typical meal timing habits. All your responses will be kept confidential. For each question, consider how often/week in the past two week did you do it. Please respond by choosing the frequency that best reflects your experience: - Less than 3 times/week -equal to or more than 3 times/week.

Please answer honestly based on your own experiences.

1- During the past two weeks “On average, how many times a week did you skip breakfast?

- Less than 3 times/week - equal to or more than 3 times/week

2- During the past two weeks “On average, how many times per week do you eat “a late dinner before bed", (i.e. between 9 p.m. and 6 a.m.)?

- Less than 3 times/week - equal to or more than 3 times/week

3- During the past two weeks “How frequently a week do you eat snacks after dinner?”

- Less than 3 times/week - equal to or more than 3 times/week

----
