# Supplementary material for: Shiftwork and insulin resistance in professional drivers: exploring the association using non-insulin-based surrogate measures
Source: BMC Public Health. 2025 Jan 16;25:191. doi: 10.1186/s12889-024-21243-9 (PMC11740691; doi:10.1186/s12889-024-21243-9)
Supplement: Supplementary file 2 — Supplementary Material 2 [file 12889_2024_21243_MOESM2_ESM.docx]

**Supplementary File**

**Table S1. Variables predicting elevated NIRS using external cut-off points: Results of multivariate analyses (N = 380).**

| **IR Surrogates** | **Predictors** | **OR** | **95% CI** | ***p*-value** | **Model fit** |
| --- | --- | --- | --- | --- | --- |
| **TyG** | Shift work (vs. day work) | 2.34 | 1.10 – 4.99 | 0.028* | **Overall accuracy:** 86.3%  **Hosmer & Lemeshow Test:** Χ^2^=9.82, df=8, *p*-value=0.278  **Nagelkerke R Square:** 0.508  **AUC**=0.902 |
|  | Age (years) | 1.02 | 0.84 – 1.11 | 0.616 |  |
|  | Physical activity (Moderately active vs. inactive) | 1.07 | 0.52 – 2.20 | 0.848 |  |
|  | Physical activity (Very active vs. inactive) | 1.52 | 0.48 – 4.75 | 0.476 |  |
|  | Overweight/obese (vs. normal weight) | 2.27 | 1.16 – 4.44 | 0.017* |  |
|  | Work duration (years) | 0.90 | 0.80 – 1.01 | 0.062 |  |
|  | Poor meal timing habits (single/multiple vs. none) | 12.63 | 4.11 – 38.79 | 0.000*** |  |
|  | PSS-10 (severe vs. low stress) | 1.04 | 0.98 – 1.09 | 0.232 |  |
|  | Moderate/severe insomnia (vs. No clinically significant/Sub-threshold) | 3.86 | 1.88 – 7.90 | 0.000*** |  |
|  | *Model Constant* | 0.34 | - | 0.541 |  |
|  | **Predictors** | **OR** | **95% CI** | ***p*-value** |  |
| **TyG-BMI** | Shift work (vs. day work) | 4.31 | 2.47 – 7.52 | 0.000*** | **Overall accuracy:** 72.9%  **Hosmer & Lemeshow Test:** Χ^2^=8.99, df=8, *p*-value=0.343  **Nagelkerke R Square:** 0.322  **AUC**=0.781 |
|  | Age (years) | 1.06 | 1.00 – 1.12 | 0.043* |  |
|  | Physical activity (Moderately active vs. inactive) | 1.31 | 0.75 – 2.28 | 0.348 |  |
|  | Physical activity (Very active vs. inactive) | 1.62 | 0.71 – 3.69 | 0.251 |  |
|  | Work duration (years) | 1.03 | 0.95 – 1.13 | 0.419 |  |
|  | Poor meal timing habits (single/multiple vs. none) | 1.16 | 0.62 – 2.17 | 0.651 |  |
|  | PSS-10 (severe vs. low stress) | 3.53 | 2.01 – 6.18 | 0.000*** |  |
|  | Moderate/severe insomnia (vs. No clinically significant/Sub-threshold) | 2.35 | 1.22 – 4.51 | 0.011* |  |
|  | *Model Constant* | 0.003 | - | 0.000*** |  |
|  | **Predictors** | **OR** | **95% CI** | ***p*-value** |  |
| **TG/HDL-C** | Shift work (vs. day work) | 0.82 | 0.38 – 1.75 | 0.600 | **Overall accuracy:** 81.1%  **Hosmer & Lemeshow Test:** Χ^2^=8.39, df=8, *p*-value=0.396  **Nagelkerke R Square:** 0.452  **AUC**=0.860 |
|  | Age (years) | 0.99 | 0.92 – 1.07 | 0.877 |  |
|  | Average monthly income (>12k LE vs. ≤12kLE) | 2.29 | 1.10 – 4.77 | 0.026* |  |
|  | Physical activity (Moderately active vs. inactive) | 1.37 | 0.74 – 2.54 | 0.323 |  |
|  | Physical activity (Very active vs. inactive) | 0.81 | 0.33 – 2.01 | 0.656 |  |
|  | Overweight/obese (vs. normal weight) | 2.37 | 1.33 – 4.23 | 0.003** |  |
|  | Work duration (years) | 0.96 | 0.87 – 1.06 | 0.379 |  |
|  | Poor meal timing habits (single/multiple vs. none) | 2.42 | 1.03 – 5.66 | 0.042* |  |
|  | PSS-10 (severe vs. low stress) | 1.41 | 0.69 – 2.87 | 0.349 |  |
|  | Moderate/severe insomnia (vs. No clinically significant/Sub-threshold) | 4.48 | 2.44 – 8.20 | 0.000*** |  |
|  | Shift work-by- bad meal-timing habits | 3.55 | 1.01 – 12.27 | 0.048* |  |
|  | *Model Constant* | 0.44 |  | 0.591 |  |
|  | **Predictors** | OR | 95% CI | *p*-value |  |
| **METS-IR** | Shift work (vs. day work) | 0.43 | 0.13 – 1.49 | 0.184 | **Overall accuracy:** 89.2%  **Hosmer & Lemeshow Test:** Χ^2^=9.11, df=8, *p*-value=0.333  **Nagelkerke R Square:** 0.389  **AUC**=0.868 |
|  | Age (years) | 1.08 | 0.99 – 1.18 | 0.068 |  |
|  | Relevant family history (vs. none) | 1.98 | 0.98 – 4.01 | 0.056 |  |
|  | Physical activity (Moderately active vs. inactive) | 1.30 | 0.40 – 4.21 | 0.664 |  |
|  | Physical activity (Very active vs. inactive) | 0.67 | 0.18 – 2.45 | 0.541 |  |
|  | Work duration (years) | 1.07 | 0.95 – 1.19 | 0.255 |  |
|  | Poor meal timing habits (single/multiple vs. none) | 2.33 | 0.73 – 7.46 | 0.153 |  |
|  | PSS-10 (severe vs. low stress) | 0.574 | 0.16 – 2.08 | 0.398 |  |
|  | ISI score | 1.23 | 1.12 – 1.35 | 0.000*** |  |
|  | Shift work-by- severe stress | 4.60 | 0.99 – 21.32 | 0.051 |  |
|  | *Model Constant* | 0.000 |  | 0.000*** |  |

*Statistically significant at *p* < 0.05, **Statistically significant at *p* < 0.01; ***Statistically significant at *p* < 0.001.

**Table S2. Accuracy of regression model I as compared to regression model II, using ROC curve analysis.**

| **NIRS** | **AUC^a^** | | **AUC difference** | **z** | ***p*-value** |
| --- | --- | --- | --- | --- | --- |
|  | **Model I** | **Model II** |  |  |  |
| **TyG** | 0.898 | 0.902 | -0.004 | -0.185 | 0.854 |
| **TyG-BMI** | 0.809 | 0.781 | 0.028 | 0.813 | 0.416 |
| **TyG/HDL-C** | 0.853 | 0.860 | -0.007 | -0.228 | 0.820 |
| **METS-IR** | 0.826 | 0.868 | -0.042 | -1.352 | 0.176 |

^a^AUC of regression model I based on sample-based cut-off points, and regression model II based on external cut-off points.

| **a** | **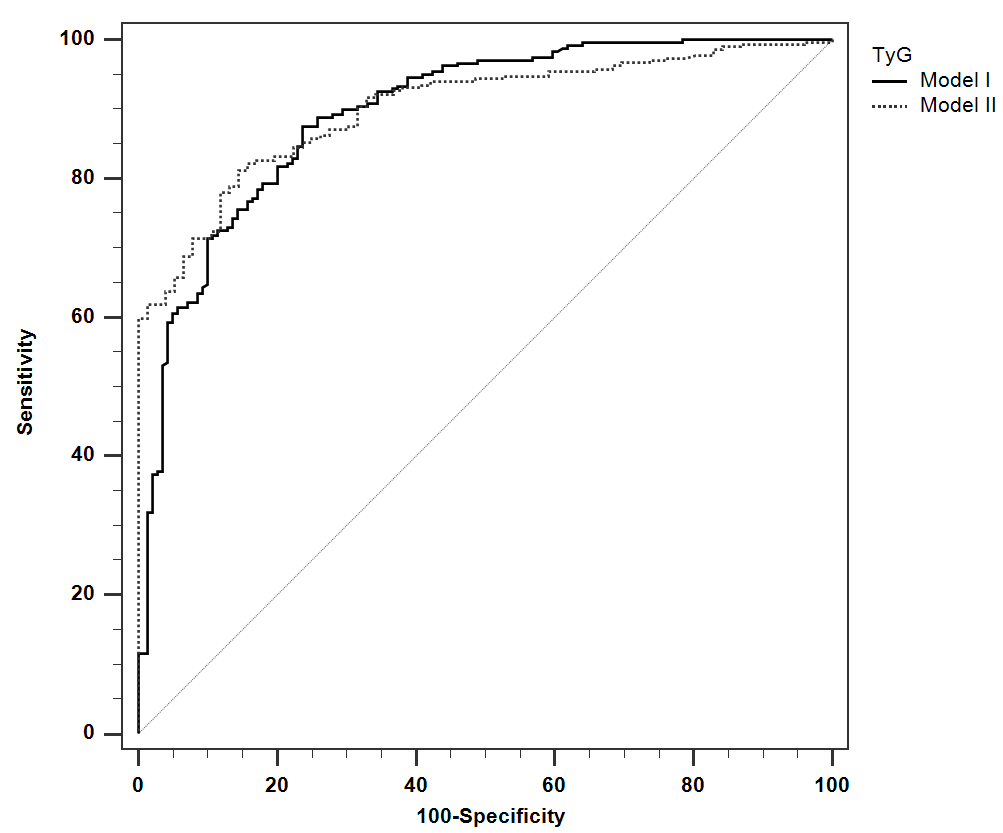** | **b** | 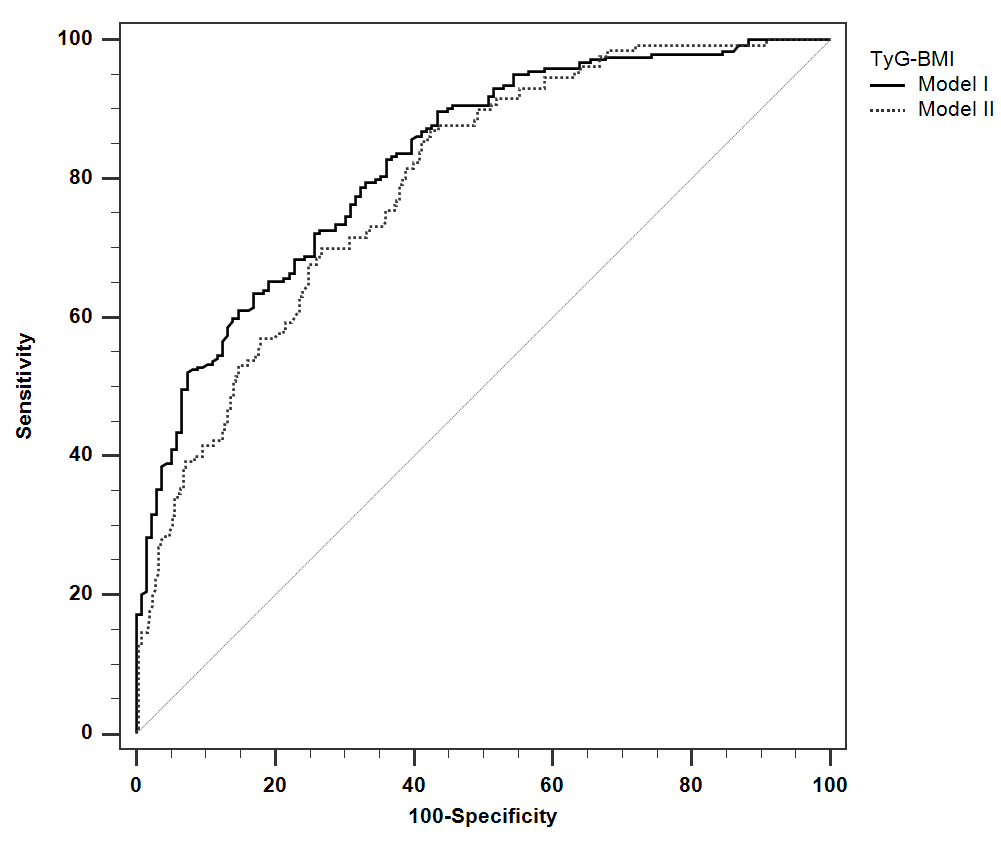 |
| --- | --- | --- | --- |
| **c** | **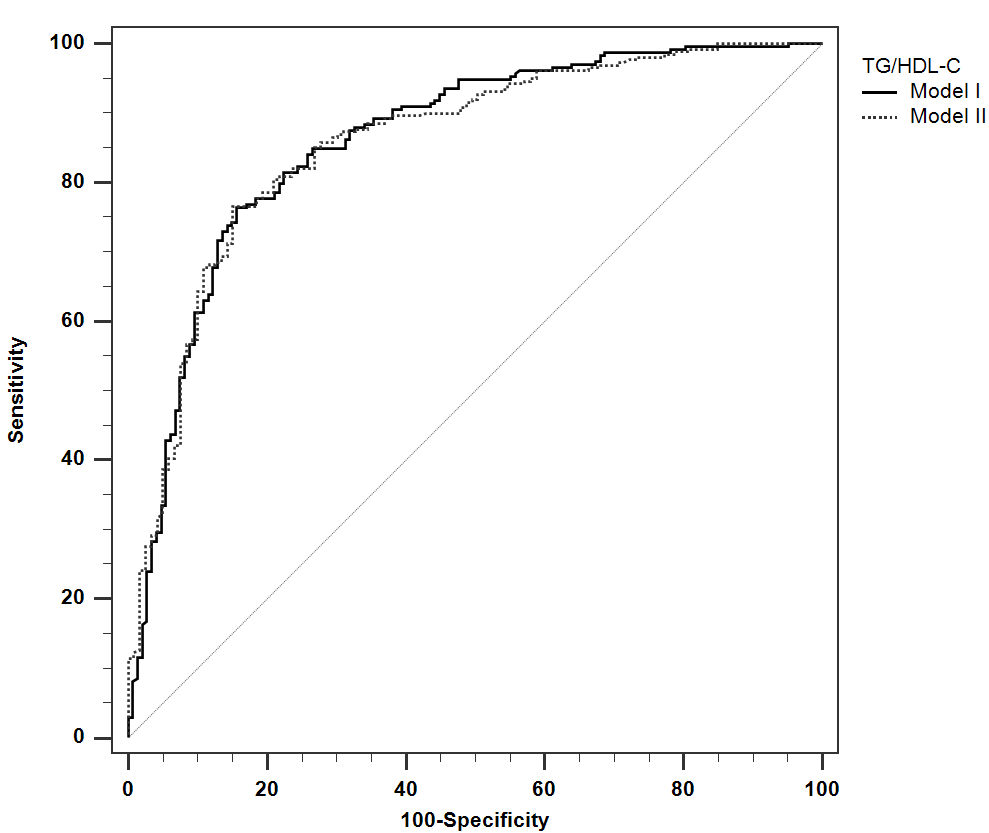** | **d** | 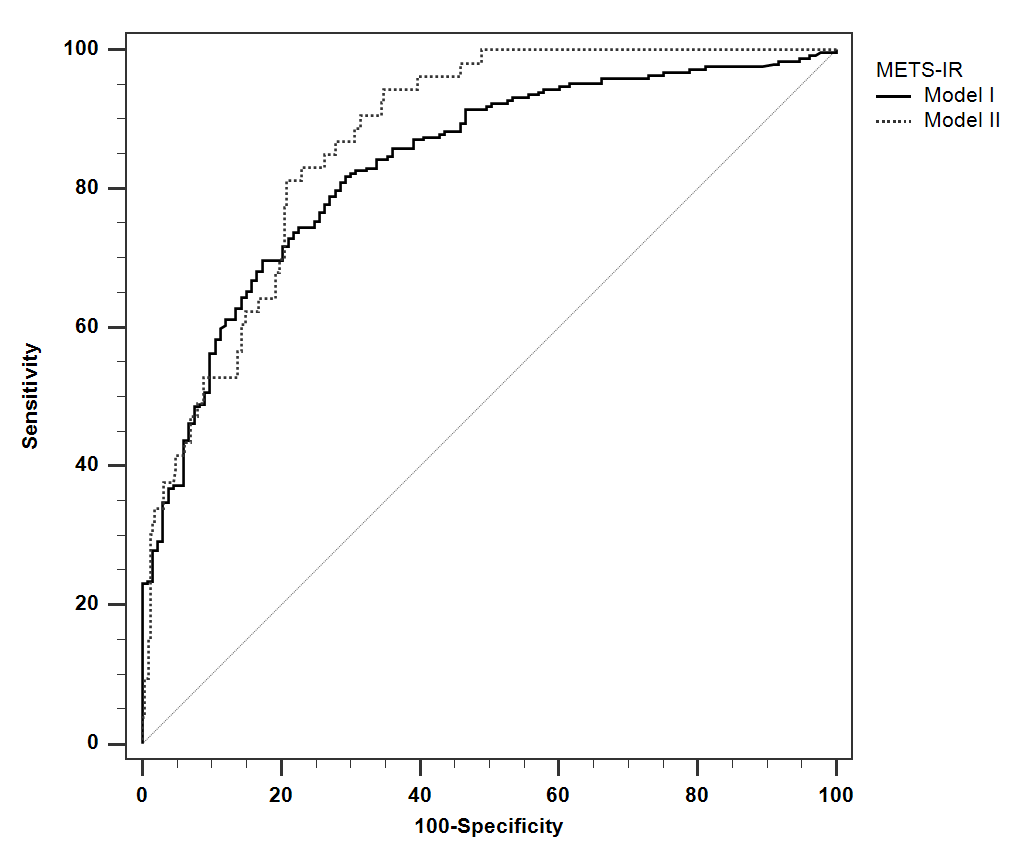 |

**Figure S1** ROC curves of regression models for predicting elevated NIRS using sample-based cut-off points in model I and external cut-off points in model II. **a** ROC curves of the regression models for predicting elevated TyG using a sample-based median (Model I) versus an external cut-off point (Model II). **b** ROC curves of the regression models for predicting elevated TyG-BMI using a sample-based median (Model I) versus an external cut-off point (Model II). **c** ROC curves of the regression models for predicting elevated TG/HDL-C using a sample-based median (Model I) versus an external cut-off point (Model II). **d** Receiver Operating Characteristic (ROC) curves of the regression models for predicting elevated METS-IR using a sample-based median (Model I) versus an external cut-off point (Model II).
