# Supplementary figures and images for: Shiftwork and insulin resistance in professional drivers: exploring the association using non-insulin-based surrogate measures
Source: BMC Public Health. 2025 Jan 16;25:191. doi: 10.1186/s12889-024-21243-9 (PMC11740691; doi:10.1186/s12889-024-21243-9)

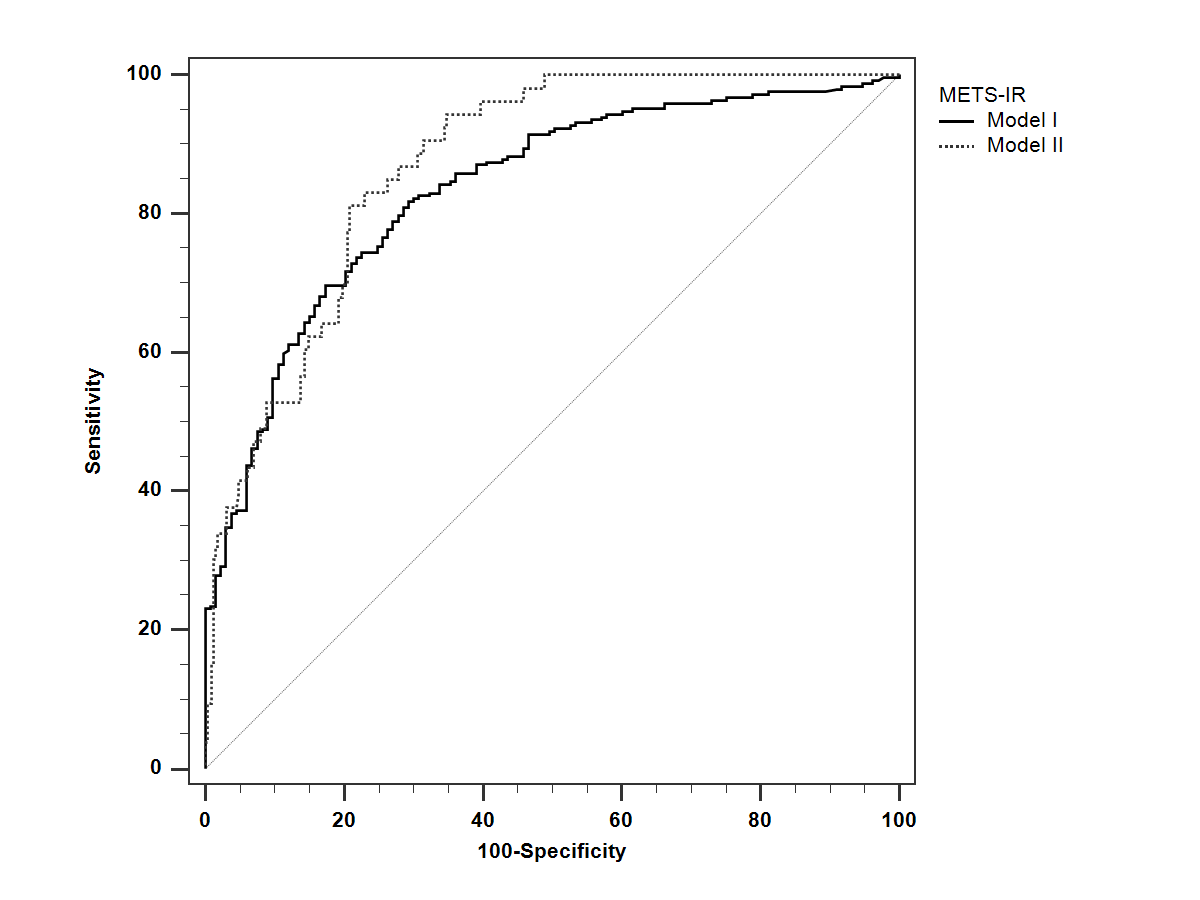

Supplement: Supplementary file 3 — Supplementary Material 3 [file 12889_2024_21243_MOESM3_ESM.tif]

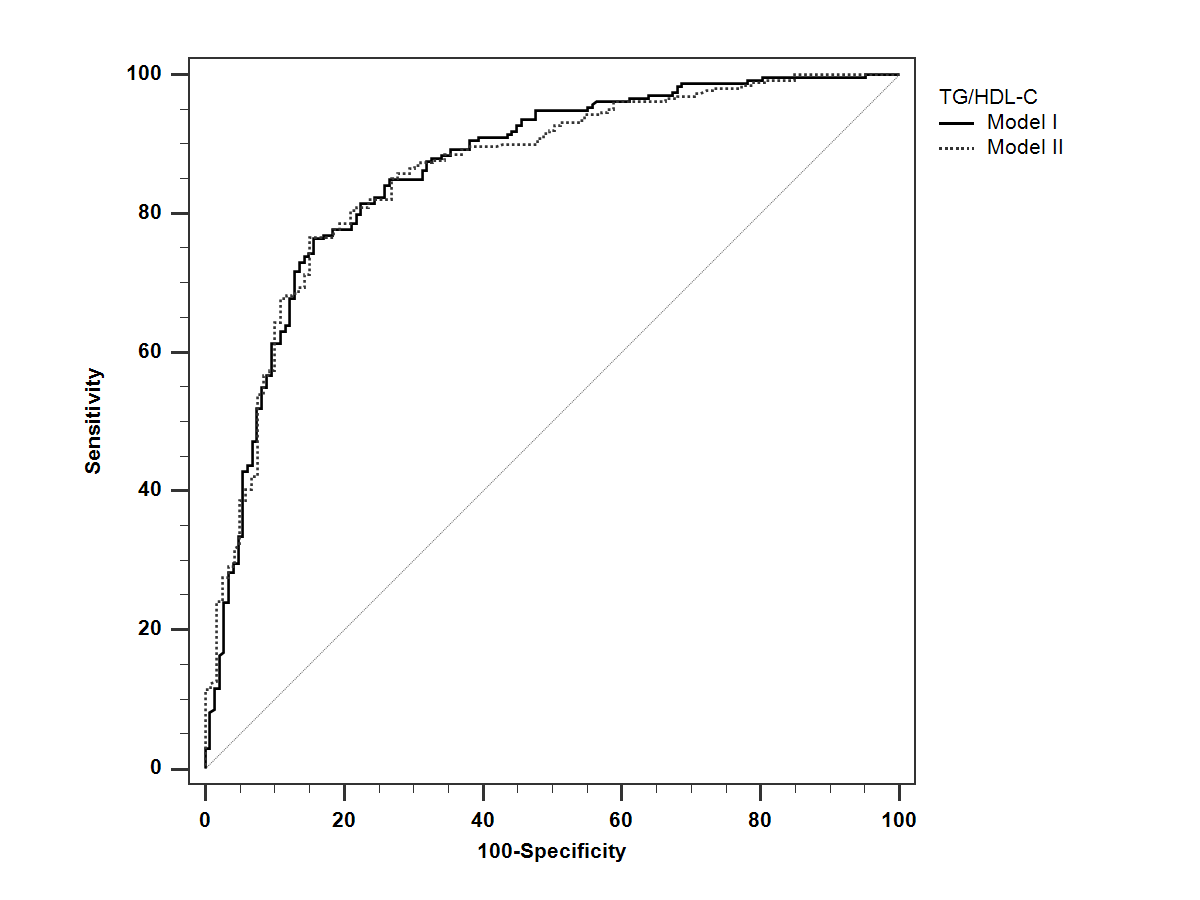

Supplement: Supplementary file 4 — Supplementary Material 4 [file 12889_2024_21243_MOESM4_ESM.tif]

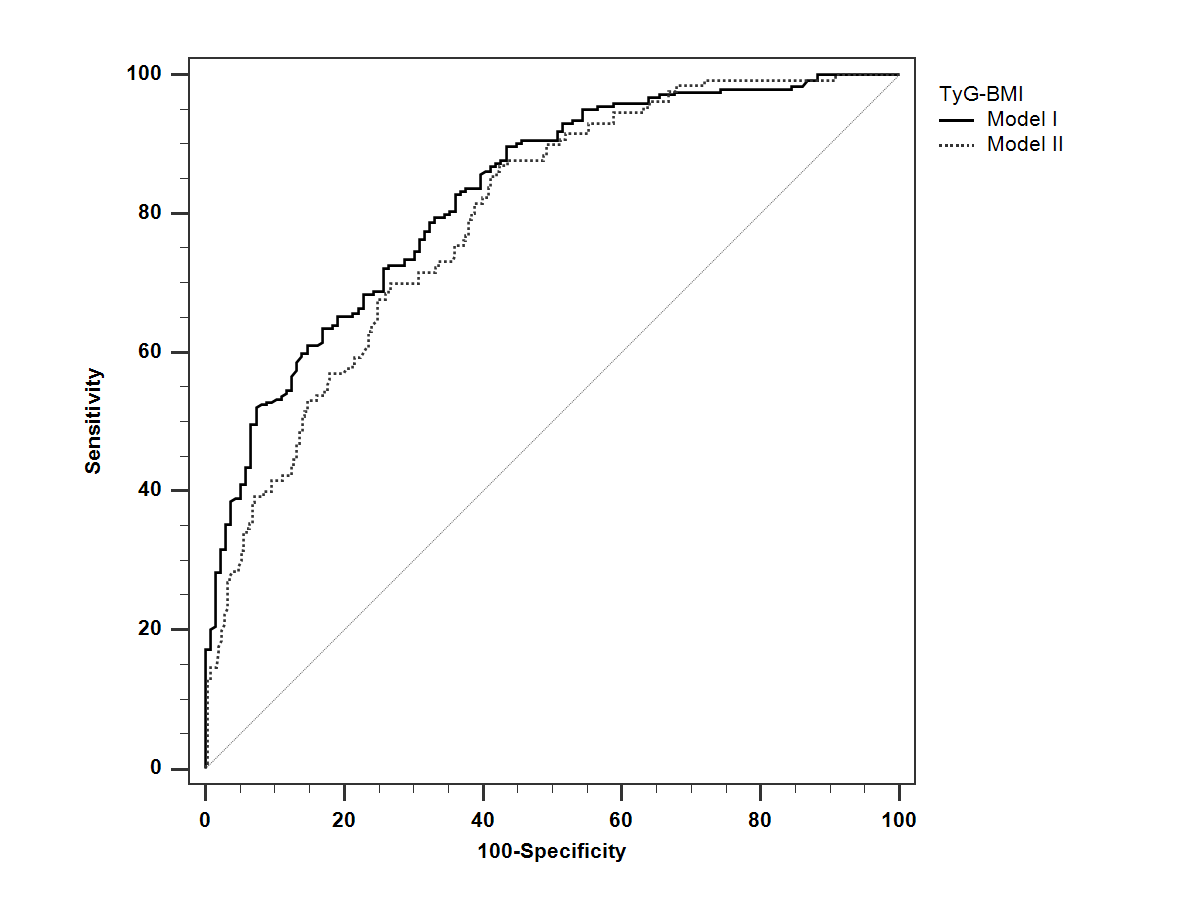

Supplement: Supplementary file 5 — Supplementary Material 5 [file 12889_2024_21243_MOESM5_ESM.tif]

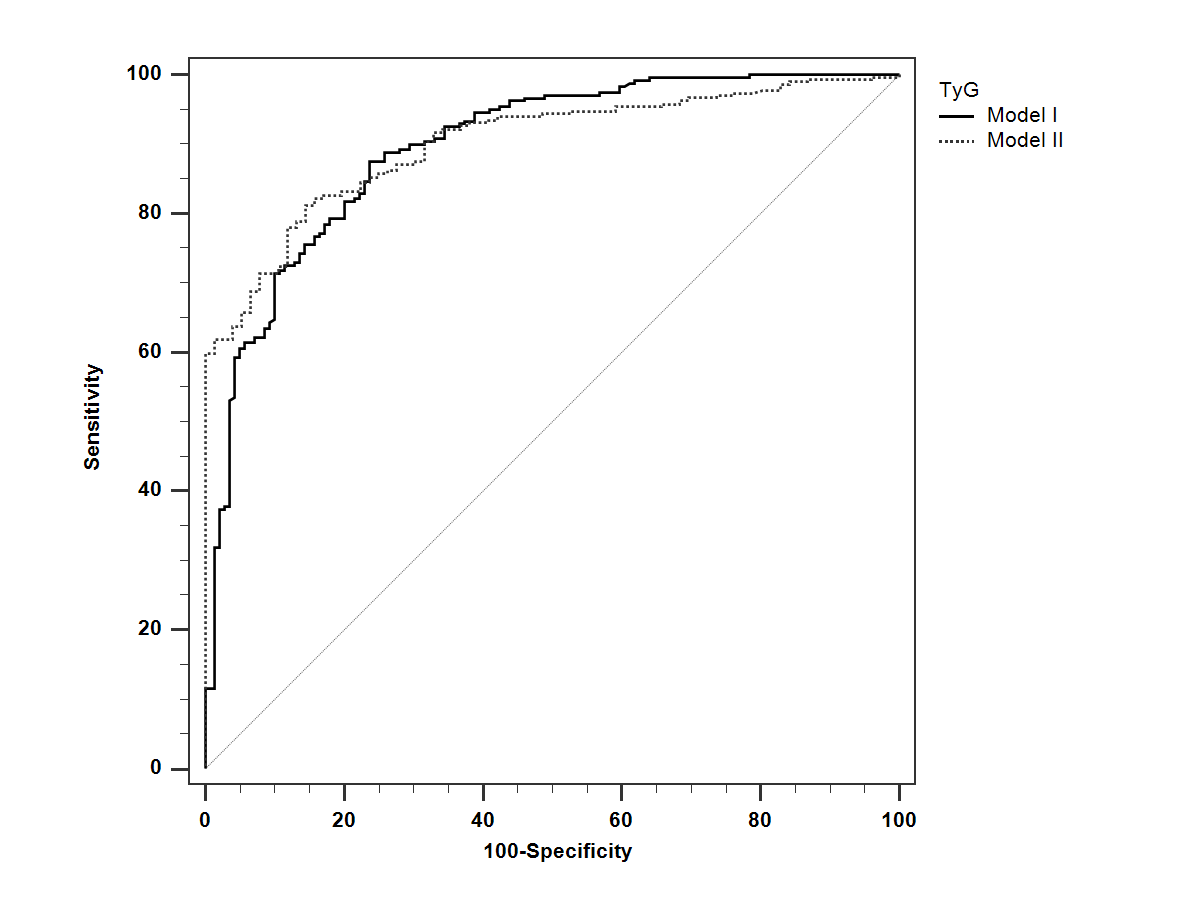

Supplement: Supplementary file 6 — Supplementary Material 6 [file 12889_2024_21243_MOESM6_ESM.tif]
